# Atypical Clinical and Diagnostic Features in Ménétrier's Disease in a Child

**DOI:** 10.1155/2011/480610

**Published:** 2011-09-21

**Authors:** Michael Chung, Jaime Pittenger, Deborah Flomenhoft, Jeffrey Bennett, Eun-Young Lee, Harohalli Shashidhar

**Affiliations:** ^1^University of Kentucky College of Medicine, 138 Leader Avenue, Lexington, KY 40506-9983, USA; ^2^University of Kentucky Children's Hospital, 800 Rose Street, Lexington, KY 40536-0298, USA; ^3^Department of Pediatrics, Kentucky Clinic, J445 740 S. Limestone, Lexington, KY 40536-0284, USA; ^4^Department of Internal Medicine, Kentucky Clinic, Room J525, 740 South Limestone, Lexington, KY 40536-0284, USA; ^5^Department of Pathology, Medical Science Building, MS117, 800 Rose St., Lexington, KY 40536-0298, USA; ^6^Division of Pediatric Gastroenterology and Nutrition, Department of Pediatrics, University of Kentucky College of Medicine, 800 Rose Street, 109, Lexington, KY 40536-0298, USA

## Abstract

Ménétrier's disease is one of the rarest protein-losing gastropathies in childhood. It is characterized clinically by non-specific gastrointestinal symptoms and edema, biochemically by hypoalbuminemia, and pathologically by enlarged gastric folds. In adults, this disease can be devastating with significant morbidity and mortality. In childhood, it is a self-limiting, transient and benign illness. Its treatment is largely supportive with total parenteral nutrition (TPN) while oral intake is encouraged. Acute onset of vomiting in healthy school age children can be initially explained by acute viral gastroenteritis. However, persistent vomiting associated with hematemesis and severe abdominal pain should warrant further work-up. This case report illustrates a self-limiting and rare cause of protein-losing enteropathy called Ménétrier's disease that presented with several variant clinical features not typically described in association with this entity.

## 1. Introduction

Ménétrier's disease is an uncommon cause of protein-losing gastropathy in children characterized by hypertrophy of gastric folds affecting the gastric body [1]. Symptoms at onset include nausea and vomiting, with profound hypoalbuminemia developing within a few days. The clinical course in children is distinct from adult-onset disease. The relative rarity of the diagnosis in combination with symptoms that mimic nonspecific gastroenteritis at onset may delay early recognition. This paper describes several variant clinical features not typically described in association with this entity.

## 2. Patient Presentation

A 7-year-old African-American female was readmitted to our institution 4 days after discharge from the hospital with recurrent non-bloody, nonbilious emesis. She did not have diarrhea, fever, or skin rash. The family did not report any sick contacts. 

She had spent ten days in the hospital during the first admission with an episode of hematemesis, abdominal pain, persistent nausea, and vomiting along with poor intake requiring parenteral nutrition (PN) for 3 days. At the time of discharge, she was tolerating a partial normal diet with no emesis. The hospital course had been complicated by development of gluteal abscess due to MRSA infection that was drained along with two days of clindamycin intravenous administration. 

**Figure 1:**
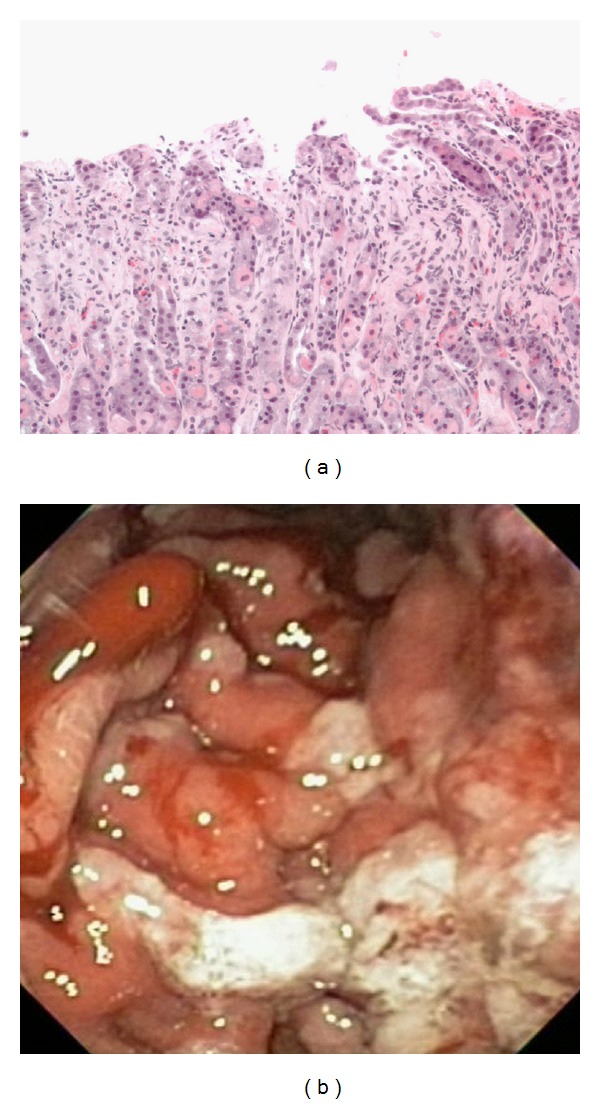
First EGD: erosion and mild eosinophilia without prominent foveolar hyperplasia (a); Endoscopic appearance of severe gastropathy (b).

An esophagogastroduodenoscopy (EGD) during the first admission had revealed severe gastropathy with thickened and friable mucosal folds in the gastric body and numerous gastric erosions with evidence of recent bleeding. Tissue and stool viral cultures had not yielded any viral growth. The biopsy showed erosions and mild eosinophilia with absent *H. pylori*. Despite the endoscopic appearance of severe gastropathy, the biopsy did not reveal foveolar hyperplasia as is typical of Ménétrier's (Figure 1). 

Laboratory evaluation during the first hospital admission included a normal complete blood count and comprehensive metabolic panel but two positive tests for stool occult blood. Urine analysis showed mild ketonuria. Hypoalbuminemia (1.8 mg/dL) was present by the third day of hospital stay. 

The child had a long-standing history of frequent vomiting and hypersensitivity reactions to certain foods and had received several courses of immunotherapy. Mother reported that the child consciously restricted her intake to a diet that consisted of bland foods such as plain pasta and bread and showed avoidance of dairy products. Despite recent weight loss, her BMI was at the 50th percentile. 

Physical examination revealed a well-developed, but ill-appearing child. She was afebrile with stable vital signs. General physical examination was notable for mild periorbital edema and conjunctival pallor. Systemic examination was unremarkable including absence of any abdominal findings. Peripheral edema was absent. 

Laboratory evaluation at second admission revealed normal complete blood count, mild ketonuria, and a urine's specific gravity of >1030. A mild lipase elevation (62) was present, and serum albumin of 1.7 had further decreased to 1.4 by the third day along with a normal prealbumin. Serum gastrin was not measured. Other studies over the course of the hospital stay included low immunoglobulin G and M levels (IgG < 200 mg/dL, IgM 27 mg/dL). By the time of discharge, immunoglobulins were normal with the exception of improved but low IgG level (350 mg/dL). The serum albumin had remained low at 1.5. A fecal *α*-1-antitrypsin level was elevated at >1.33, consistent with a protein-losing enteropathy. Additional testing included normal serum cortisol level and negative tissue transglutaminase assay. 

**Figure 2:**
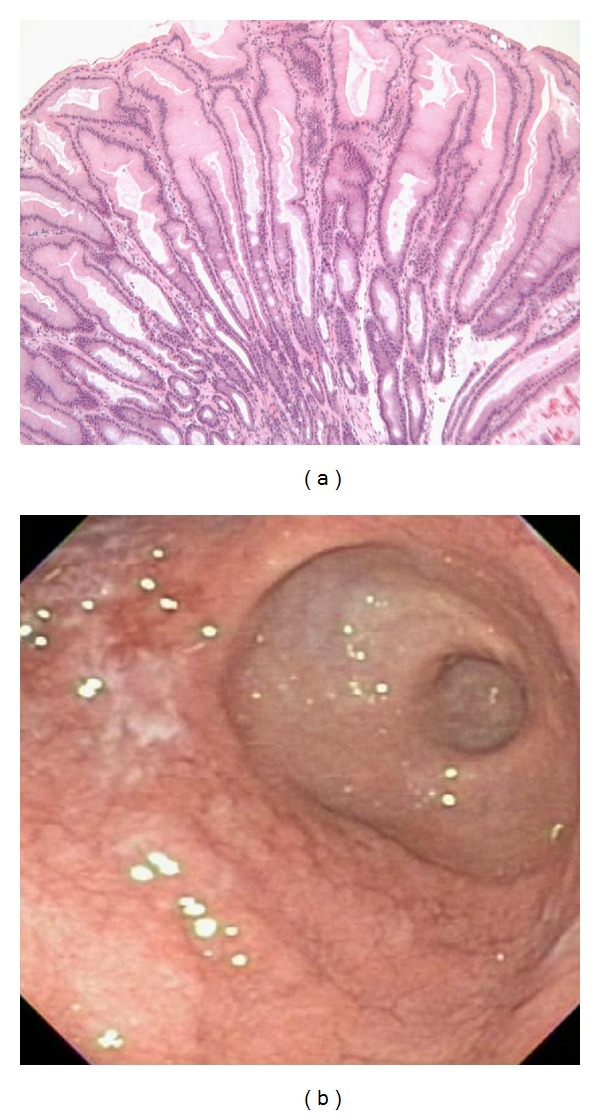
Second EGD: prominent foveolar hyperplasia on gastric biopsy (a). Improved endoscopic appearance; note sparing of antrum (b).

 A repeat endoscopy was performed along with a colonoscopy during the second admission. Endoscopy showed edematous and thickened folds in the gastric body with a normal-appearing gastric fundus and antrum along with partial resolution of mucosal friability and erosions seen previously. The gastric biopsy revealed erosions, mild eosinophilia, along with characteristic prominent foveolar hyperplasia (Figure 2). Mild eosinophilia was found in duodenum, colon, and terminal ileum. A tissue culture again failed to yield any viral growth. Ultrasound-guided paracentesis of modest pelvic fluid showed a transudate and yielded no microbial growth. 

**Figure 3:**
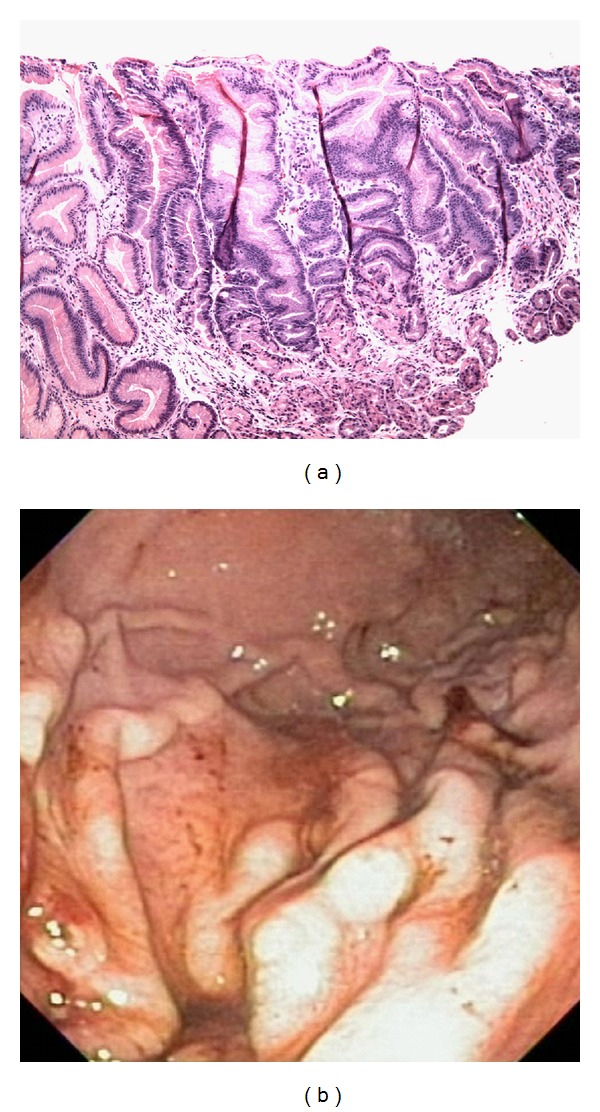
Third EGD shows persisting foveolar hyperplasia (a), erosions but improved reactive gastropathy (b).

She continued to improve over the course of the admission, with decreased abdominal pain and emesis “back to baseline.” The plasma albumin level remained low with the nadir of 1.4 g/dL along with poor appetite and intake. PN was initiated on the day 3 of hospital admission, with subsequent clinical improvement. At the time of discharge, 12 days after admission, she was tolerating 50% of her required caloric intake and registered a weight gain of 1 kg. Serum albumin at discharge was low normal at 2.7 and increased to 3.7 at follow-up 4 weeks later. A follow-up endoscopy one month later showed significant resolution of gastric hypertrophy with absent erosions and only focal foveolar hyperplasia (Figure 3).

## 3. Discussion

As of 2008, approximately 50 to 60 cases of pediatric Ménétrier's disease are reported in the literature [2]. The child described here exhibited significant protein-losing enteropathy with profound hypoproteinemia. Other causes of protein-losing enteropathy relevant to this case include eosinophilic gastroenteritis, gastric lymphoma, celiac disease, hypertrophic gastropathy, in association with *H. pylori* infection, and Crohn's disease [2–5]. Published case series favor a male and Caucasian preponderance for Ménétrier's disease [4, 6–8].

Ménétrier's disease in children resembles the adult-onset in its presentation with nausea, vomiting, and abdominal pain [9]. Diagnosis is typically made by presence of thickened mucosal folds, during an upper gastrointestinal contrast study or upper gastrointestinal endoscopy, or by ultrasound or CT imaging [9–11]. In adults, Ménétrier's disease usually follows a chronic, unremitting course leading to significant morbidity and mortality due to ongoing protein loss and life-threatening gastrointestinal hemorrhage [3, 4]. Ménétrier's disease in children appears to be generally benign and self-limited, with spontaneous resolution and recovery within 5 months [5, 12, 13]. An adult case series described vomiting and edema in 50% of the group [14] with a mean serum albumin at presentation of 1.8 g/dL (range 1.5-2.5 g/dL). Generalized edema is less common being present in less than 25% [9]. In contrast, peripheral edema is reported in up to 90% of children [3, 6]. Ascites and pleural effusion can result from pediatric Ménétrier's disease with severe protein loss [4]. The child described did not present with clinical ascites or peripheral edema typically present in children with hypoalbuminemia. Hematemesis and erosive gastropathy were also present in the child; anemia and bleeding are less common features described in only a few papers [7, 9]. 

Histopathology of Ménétrier's disease is similar in adults and children with hypertrophy of gastric glands, cystic dilatation of glands deep in the mucosa, an increase in mucin secreting cells, inflammatory cell infiltration, and thickened hyperplastic mucosa [7, 13]. The first endoscopic biopsy in our case showed absence of glandular dilation or hypertrophy typical for Ménétrier's disease, confirmed on a retrospective comparative review of serial endoscopic biopsies by a single pathologist. The biopsy from the second endoscopy did reveal characteristic histological changes, including foveolar hyperplasia. This discrepancy may relate to sampling error or timing of endoscopy, being performed earlier in the course of the disease. Moreover, endoscopy has not always been utilized to make the diagnosis in previous published reports. Clinicopathologic correlation is necessary to make a diagnosis of Ménétrier's disease, endoscopic biopsy alone being insufficient.

Ménétrier's disease may be confused with eosinophilic gastroenteritis as peripheral eosinophilia can be found in about 66.7% of reported cases [13]. However, peripheral eosinophilia in eosinophilic gastroenteritis ranges from 13–55%, higher than that usually seen in Ménétrier's disease [15]. Burns and Gay postulated an allergic cause as the basis of peripheral eosinophilia and eosinophilic mucosal infiltrate [16]. Eosinophilic gastroenteritis may also present with predominant or exclusive serosal involvement; peritoneal fluid analysis in this instance did not show eosinophils. The gastric and duodenal biopsy from this child revealed mild to moderate eosinophilic infiltration. It is unclear if this is part of Ménétrier's disease histopathology or suggests underlying eosinophilic enteritis, given a history of long-standing vomiting with certain food triggers and food avoidance. A subsequent food allergy evaluation did not reveal presence of food allergens. 

A definitive cause of Ménétrier's disease is still unknown and widely debated [9]. Speculated etiology includes chemical irritants, toxins, dietary factors, neuroemotional, endocrinologic, or immunologic abnormalities, allergic processes, and autoimmune disorders. The best described association is with CMV infection. One study reported that CMV infection was associated in 19 of 27 children with Ménétrier's disease [17]. Another review of children with Ménétrier's disease found an association with CMV infection in 26 of 56 cases. Gastric biopsy showed characteristic CMV inclusion bodies in 16 of those [9]. CMV infections are relatively uncommon in adults with Ménétrier's disease with the exception of immunocompromised hosts. In contrast, *H. pylori* infection is more prevalent in Ménétrier's in adults while uncommon in children [12, 18, 19]. *H. pylori* was absent in all three endoscopic biopsies in the child described. 

A familial form of protein-losing enteropathy with autosomal dominant inheritance has been reported [20]. TGF-*α*, a ligand to EGFR, may play a role in the pathogenesis of Ménétrier's disease due to its local growth-stimulating effect via EGFR binding, leading to foveolar hyperplasia and hypertrophic gastropathy in mice [21, 22]. This specific mediator is found to be over-expressed in adults and children with Ménétrier's [23–25]. Suggested pathogenic mechanism of mucosal damage caused by CMV infection may involve the production of abnormal local TGF-*α*, which stimulates cell proliferation of gastric mucosa, inhibits gastric secretion, and enhances mucus secretion [26]. 

This African-American female child with Ménétrier's disease exhibited several atypical features. Absence of edema, initial presentation with erosive gastropathy, as well as presence of mild to moderate mucosal eosinophilia in the face of long-standing vomiting with certain food triggers confounded the diagnosis. Ménétrier's features were only discovered on subsequent endoscopic biopsies. Infectious agents including CMV and *H. pylori* were absent on repeated testing. This illustrates the importance of maintaining an index of suspicion for this diagnosis and highlights upper endoscopy with adequate biopsies as a useful diagnostic test for Ménétrier's disease.

##  Disclosure

The authors have indicated they have no financial relationships relevant to this paper to disclose.

## Abbreviations

EGD: Esophagogastroduodenoscopy
PN: Parenteral nutrition
CMV: Cytomegalovirus
EGFR: Epidermal growth factor receptor
GI: Gastrointestinal.
